# Plant quality declines as CO_2_ levels rise

**DOI:** 10.7554/eLife.03233

**Published:** 2014-05-28

**Authors:** Hans-Joachim Weigel

**Affiliations:** 1 **Hans-Joachim Weigel** is at the Thünen Institute of Biodiversity, Johann Heinrich von Thünen Institute, Braunschweig, Germanyhans.weigel@ti.bund.de

**Keywords:** elevated CO_2_, zinc, iron, ionome, crops, human nutrition, None

## Abstract

There is concern that crop plants are becoming less nutritious as the levels of carbon dioxide in the atmosphere increase.

**Related research article** Loladze I. 2014. Hidden shift of the ionome of plants exposed to elevated CO_2_ depletes minerals at the base of human nutrition. *eLife* **3**:e02245. doi: 10.7554/eLife.02245

**Image** Elevated levels of CO_2_ increase the concentration of carbon (red) but reduce the concentration of many different minerals (blue) in plants

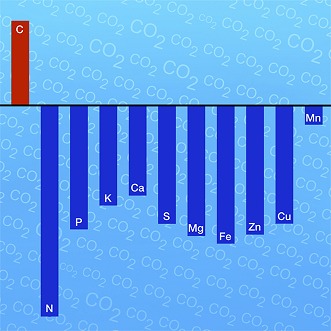

The rapid rise in the concentration of carbon dioxide, or CO_2_, in the atmosphere, which has occurred largely since the Industrial Revolution, is the most prominent cause of climate change. Moreover, atmospheric CO_2_ levels are predicted to double between now and the end of this century (IPCC, 2013). Most plants respond to elevated CO_2_ concentrations by growing faster, and it is possible that this enhanced growth might counteract some of the negative impacts of climate change on global food production, such as decreased agricultural output due to an increased frequency of floods and droughts.

However, alongside this enhanced growth, plants that are exposed to elevated levels of CO_2_ frequently show changes in the chemical composition of their leaves, stems, roots, fruits and tubers. While these effects have been known for some time (Porter and Grodzinski, 1984; Manderscheid et al., 1995), they have not been assessed in a systematic and comprehensive manner. Whether or not these changes in the chemical composition of food crops have wider implications for human nutrition is a matter of debate.

To address this question, Irakli Loladze of the Catholic University of Daegu has systematically collected and evaluated existing information on the effects of elevated levels of CO_2_ on plant tissues, notably the effects on minerals and trace elements that are important for human health, such as calcium, zinc, and iron. This meta-analysis—which is published in *eLife*—analysed more than 7500 observations that cover 130 different plant species and crop varieties (Loladze, 2014). Based on this unprecedentedly large dataset, Loladze was able to show that, when averaged across very different plant and tissue types, experimental approaches and locations, elevated CO_2_ reduced the overall mineral content of plants by about 8%. At the same time, elevated CO_2_ was shown to strongly increase the ratio of soluble carbohydrates (starch and sugars) to proteins.

Making general conclusions about the effects of elevated CO_2_ on the composition of plant tissues derived from existing studies is difficult. Although decreased concentrations of nitrogen, protein, and other minerals have been previously observed, the size of these effects is often small and thus difficult to confirm with small sample sizes. Furthermore, not all elements are affected in the same way in different plant species and experiments. Despite these caveats, Loladze's meta-analysis shows a remarkable consistency in the effects of elevated CO_2_ on the mineral concentrations across a wide range of plant species, including food crops.

There are less data available for edible parts of major food crops, and a general lack of data for other important food crops, such as maize, banana and cassava. Nevertheless, Loladze could show diminished mineral concentrations in edible parts of food crop plants from existing information. Further, he argues that the observed changes to the mineral content of food crops might exacerbate the problem of ‘hidden hunger’; that is, a person's diet can be deficient in minerals even if they consume enough calories. This type of malnutrition is common, particularly in developing countries, as many people eat only a limited number of staple crops, and do not eat enough mineral-rich foods—such as fruits, vegetables, dairy and meats. Loladze also speculates that the changes in the carbohydrate to protein ratio caused by elevated CO_2_ might contribute to the obesity epidemic.

In independent work, Samuel Myers of Harvard and co-workers have studied the effects of elevated CO_2_ on six food crops (Myers et al., 2014). They found that zinc and iron levels were diminished in the grains of wheat, rice and soybean that were grown in atmospheres containing the levels of CO_2_ that may be reached by the middle of this century.

Both Loladze and Myers et al. note that while many crops can photosynthesise more when they are grown under elevated CO_2_ levels, the photosynthesis of some crops (for example maize) is not directly influenced by elevated CO_2_. However, both types of crops are known to lose less water from their tissues when grown at elevated CO_2_, and thus the stimulation of photosynthesis and/or the improved water economy may contribute to the overall increase in growth. In contrast, the possible causes for the changes in plant tissue composition remain less clear. Previous studies have discussed the potential for a ‘dilution effect’ due to the surplus of carbohydrates, or a reduced plant uptake of minerals from the soil due to reduced water flow through the plant (McGrath and Lobell, 2013).

Although the exact mechanism remains unclear, the evidence suggests that an increase in CO_2_ levels is likely to be detrimental for human nutrition. Beyond food crops, these findings could also have implications for the quality of animal feed and for the overall element content of natural environments. Finally, there is a clear need for further research (Figure 1) that is specifically targeted to address the impact of climate change on the quality of food crops grown around the world.

**Figure 1.**
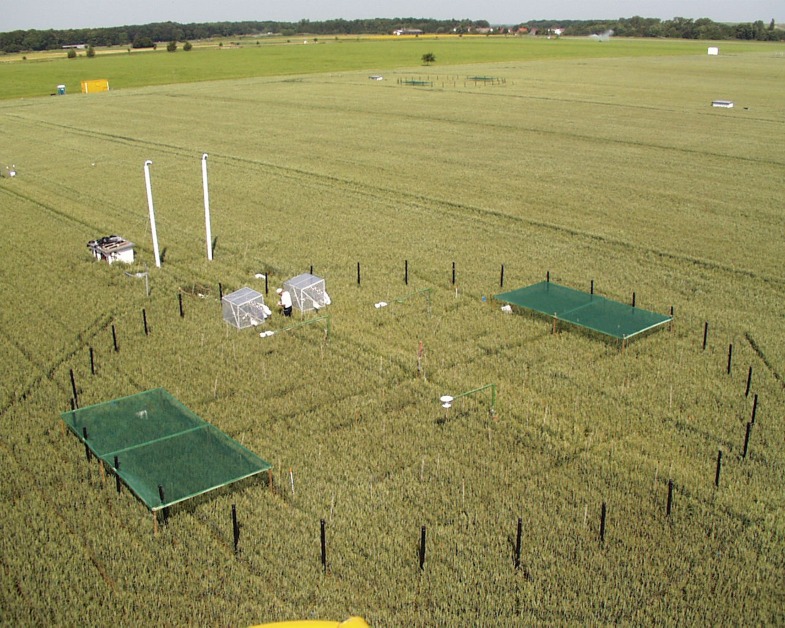
Exploring the impact of elevated CO_2_ concentrations on plant growth. In this free air carbon dioxide enrichment (FACE) experiment in Braunschweig, Germany, winter wheat plants inside the black posts are exposed to elevated levels of CO_2_ as they grow. These experiments allow researchers to simulate the effects of future atmospheric CO_2_ concentrations on plant growth under real field conditions; however data derived from these experiments are scarce.
